# The different stimulation durations of transcranial direct current stimulation for Parkinson’s disease: a systematic review and network meta-analysis

**DOI:** 10.3389/fnagi.2026.1849992

**Published:** 2026-06-10

**Authors:** Xiaojiang Yi, Yan Liang, Yuhui Wang, Dezhi Chen, Jing Huang, Yunrui Zhang

**Affiliations:** Department of Neurology, West China Hospital, Sichuan University, Chengdu, Sichuan, China

**Keywords:** network meta-analysis, Parkinson’s disease, stimulation duration, systematic review, transcranial direct current stimulation

## Abstract

**Background:**

Transcranial direct current stimulation (tDCS) holds promise for the treatment of motor symptoms in Parkinson’s disease (PD). However, the optimal stimulation duration within a single session remains unclear.

**Objective:**

This network meta-analysis (NMA) aimed to compare the relative effects of different single-session tDCS durations (13–30 min, 2 mA) on motor symptoms in patients with Parkinson’s disease.

**Methods:**

We systematically searched PubMed, Embase, Cochrane Library, and Web of Science up to May 7, 2026 for randomized controlled trials (RCTs) that compared active anodal tDCS at 2 mA with sham stimulation in patients with PD. The primary outcome was the Unified Parkinson’s Disease Rating Scale Part III (UPDRS-III), and the secondary outcome was the Timed Up and Go (TUG) test. Risk of bias was assessed using the ROB 2.0 tool, and the certainty of evidence was evaluated using the GRADE approach. A frequentist network meta-analysis was performed.

**Results:**

Sixteen RCTs (414 patients) were included. All networks were open-loop. Compared to sham, all active tDCS (2 mA, with durations of 13, 15, 20, 25, and 30 min) showed numerical improvements in UPDRS-III and TUG (0.35–1.03 s), but none reached statistical significance (all *p* > 0.05). However, probability ranking showed that 2 mA + 15 min tDCS had the highest probability of being optimal for improving UPDRS-III, while 2 mA + 13 min tDCS was most likely to be optimal for reducing TUG time. Owing to sparse indirect evidence, no definitive efficacy ranking can be made, and these rankings are descriptive only.

**Conclusion:**

No single-session tDCS duration between 13 and 30 min at 2 mA was statistically superior to sham for improving motor function in PD. The current limited evidence suggests that varying stimulation time alone within this range does not decisively impact outcomes. Our findings highlight the need for multidimensional optimization of tDCS and further high-quality RCTs.

**Systematic review registration:**

https://www.crd.york.ac.uk/PROSPERO/view/CRD42024599000.

## 1 Introduction

Parkinson’s disease (PD) is the second most prevalent neurodegenerative disease worldwide. The prevalence, disability rate, and mortality rate of this condition are increasing rapidly, making it the fastest-growing disease burden in the nervous system (Dorsey et al., 2018a,b; Zhong and Zhu, 2022). According to global burden of disease studies, the number of PD patients is projected to rise from 11.9 million in 2021 to 25.2 million in 2050, representing an increase of approximately 112% (Su et al., 2025). This trend is likely to result in an escalating clinical and socioeconomic burden.

The management of PD relies primarily on pharmacological and non-pharmacological interventions. Although pharmacotherapy is essential for symptom control, its long-term use is complicated by motor fluctuations and limited efficacy for non-motor symptoms (Connolly and Lang, 2014). Invasive neuromodulation techniques, such as deep brain stimulation, are limited by high cost and surgical risks (Deuschl et al., 2006; Weaver, 2009). In contrast, transcranial direct current stimulation (tDCS) is a non-invasive technique that has received significant attention in PD research due to its favorable safety profile, ease of application, low cost, and compatibility with rehabilitation (Benninger and Hallett, 2015; Fregni et al., 2006).

Transcranial direct current stimulation modulates cortical neuronal excitability via a weak direct current and can influence functional connectivity within the cortico-basal ganglia-thalamic-cerebellar circuit, a core pathological network in PD (Polanía et al., 2011; Bueno et al., 2019). tDCS has shown promise in enhancing motor functions in PD, such as gait and balance, these effects may involve mechanisms including enhanced motor cortex excitability, facilitated dopamine release, and modulation of neural plasticity. However, the efficacy of tDCS is modulated by several parameters. Stimulation duration is a critical variable that determines the strength and persistence of its neurophysiological and clinical effects (Nitsche and Paulus, 2000, 2001; Monte-Silva et al., 2013; Batsikadze et al., 2013).

Existing studies exhibit substantial heterogeneity in applied durations (e.g., ranging from seven to 30 min per session). This variability contributes to inconsistent results. Crucially, no study has systematically compared the relative efficacy of tDCS with different durations, leaving the optimal stimulation duration undetermined (Lee et al., 2019; de Oliveira et al., 2021; Madrid and Benninger, 2021).

Conventional pairwise meta-analysis has limitations in addressing this key evidence gap. The present study employs network meta-analysis (NMA) instead. NMA integrates direct and indirect comparison evidence within a unified framework and enables the simultaneous comparison and ranking of the efficacy of multiple tDCS intervention with different stimulation durations. This approach is particularly valuable when direct head-to-head comparisons are lacking (Salanti et al., 2014). Through a systematic review and NMA, this study aims to comprehensively evaluate the effects of tDCS with different stimulation durations on motor function in patients with PD, as well as rank the efficacy of these interventions. The findings are expected to provide direct, high-level evidence to optimize tDCS parameters in clinical practice.

## 2 Methods

### 2.1 Design

This network meta-analysis was conducted in accordance with the Cochrane Handbook for Systematic Reviews of Interventions (Page et al., 2021) and complied with the Preferred Reporting Items for Systematic Reviews and Meta-Analyses extension for Network Meta-Analyses (PRISMA-NMA) statement (Hutton et al., 2015). The study protocol was registered in PROSPERO (registration number: CRD42024599000). The full registration protocol and study details are publicly accessible at: https://www.crd.york.ac.uk/PROSPERO/view/CRD42024599000

### 2.2 Search strategy

We systematically searched four major databases (PubMed, Web of Science, Embase, and the Cochrane Library) from their inception to May 7, 2026. The search strategy combined Medical Subject Headings (MeSH) with free-text terms, and the core structure using Boolean operators was: (Parkinson Disease OR Parkinson’s Disease OR Parkinsonism) AND (Transcranial Direct Current Stimulation OR tDCS OR transcranial electrical stimulation). Additionally, we manually screened meta-analyses, reviews, and reference lists of included studies to identify additional relevant publications. Only formally published peer-reviewed articles were included. Grey literature, preprints, dissertations, conference abstracts, trial registries, and non-English studies were not searched. The complete search strategy and results are provided in Supplementary Table S1.

### 2.3 Study selection

Inclusion criteria were defined using the PICOS framework: (1) Population: Participants diagnosed with Parkinson’s disease; (2) Intervention: active anodal tDCS, either alone or combined with conventional interventions (e.g., gait training, treadmill training, cognitive training, or neurorehabilitation); (3) Comparator: sham stimulation, either alone or combined with the same conventional interventions; (4) Outcomes: the primary outcome was motor function, assessed using the Unified Parkinson’s Disease Rating Scale Part III (UPDRS-III); the secondary outcome was functional mobility, assessed using the Timed Up and Go test (TUG; in seconds); (5) Study design: parallel-group, sham-controlled randomized controlled trials (cross-over designs were excluded).

Exclusion criteria were: (1) duplicate publications (only the publication with the most complete dataset was retained); (2) studies lacking essential baseline or outcome data that could not be obtained after contacting the corresponding authors; (3) studies irrelevant to the research topic (e.g., non-tDCS interventions, non-PD populations, or non-RCT designs); (4) studies for which full texts were unavailable and we attempted to contact the authors but could not obtain the data.

### 2.4 Outcomes

The primary outcome was motor function severity, which was assessed using the Unified Parkinson’s Disease Rating Scale Part III (UPDRS-III). The secondary outcome was functional mobility, measured by the Timed Up and Go (TUG) test (recorded in seconds).

### 2.5 Data extraction and quality assessment

Two independent reviewers (X-J Yi and Y-H Wang) performed study selection, data extraction, and methodological appraisal. Disagreements were resolved by consensus with a third reviewer (D-Z Chen). A pre-designed form was used to extract data from eligible studies, including study year, country, sample size, age, disease duration, stimulation parameters, stimulation duration, outcomes, and other relevant information.

Continuous data reported as median and interquartile range were converted to mean and standard deviations (*SD*) according to established methods (Wan et al., 2014). For each outcome, the mean change from baseline to post-intervention and its SD were extracted. When multiple post-intervention time points were reported, the first assessment immediately after the end of the intervention period (i.e., the earliest time point) was selected.

The risk of bias in randomized trials was assessed using the Cochrane Risk of Bias tool (ROB 2.0) across its five domains (Sterne et al., 2019). Any discrepancies in these assessments were resolved by consensus before analysis.

The GRADE framework was used to assess evidence certainty (Guyatt et al., 2011). Certainty was rated as high, moderate, low, or very low. Randomized controlled trials started as high-certainty evidence but could be downgraded due to risk of bias, inconsistency (e.g., *I^2^* > 50%), indirectness, imprecision, or publication bias. Upgrading was possible for observational studies with a large effect size, a dose–response gradient, or residual confounding that would attenuate the observed effect. Evidence was rated as moderate when serious limitations were present in one or two domains, and as high when no serious limitations existed across all domains.

### 2.6 Statistical analysis

All outcome parameters in this network meta-analysis were continuous variables. A frequentist network meta-analysis was performed using Stata 17.0 (StataCorp, College Station, TX, USA) with the network suite of commands.

First, a pairwise meta-analysis was performed for direct comparisons. The heterogeneity was quantified using the *I^2^* statistic. A sensitivity analysis was planned to test the robustness of the pooled results by iteratively excluding individual studies. When substantial heterogeneity (*I^2^* ≥ 50%) was detected, subgroup analyses were planned to explore potential effect modifiers, including disease duration, age, and stimulation site.

The UPDRS-III and TUG test scores were analyzed as continuous outcomes, with the mean difference (MD) serving as the effect measure, accompanied by its 95% confidence interval (CI). A fixed-effects model was used when no significant heterogeneity was present (*p* > 0.05 and *I^2^* < 50%). In the absence of such evidence, an exploratory investigation was conducted to identify potential sources of heterogeneity. When a definitive clinical explanation could not be ascertained, a random-effects model was employed for data synthesis.

Subsequently, a network meta-analysis was performed within a frequentist framework using a consistency model. A network plot of the evidence was initially constructed. When closed loops were present, global and local inconsistency tests were conducted. The consistency assumption between direct and indirect evidence was considered satisfied if the *p*-value for inconsistency exceeded 0.05, or if the 95% CI for the loop-specific inconsistency factor included zero. The results of the network meta-analysis were summarized in league tables, and the surface under the cumulative ranking curve (SUCRA) was calculated to rank the comparative efficacy of different stimulation-duration tDCS. Publication bias was assessed using funnel plots and Egger’s test. The significance level (*α*) was set at 0.05 for all statistical tests.

## 3 Results

### 3.1 Study selection

**Figure 1:**
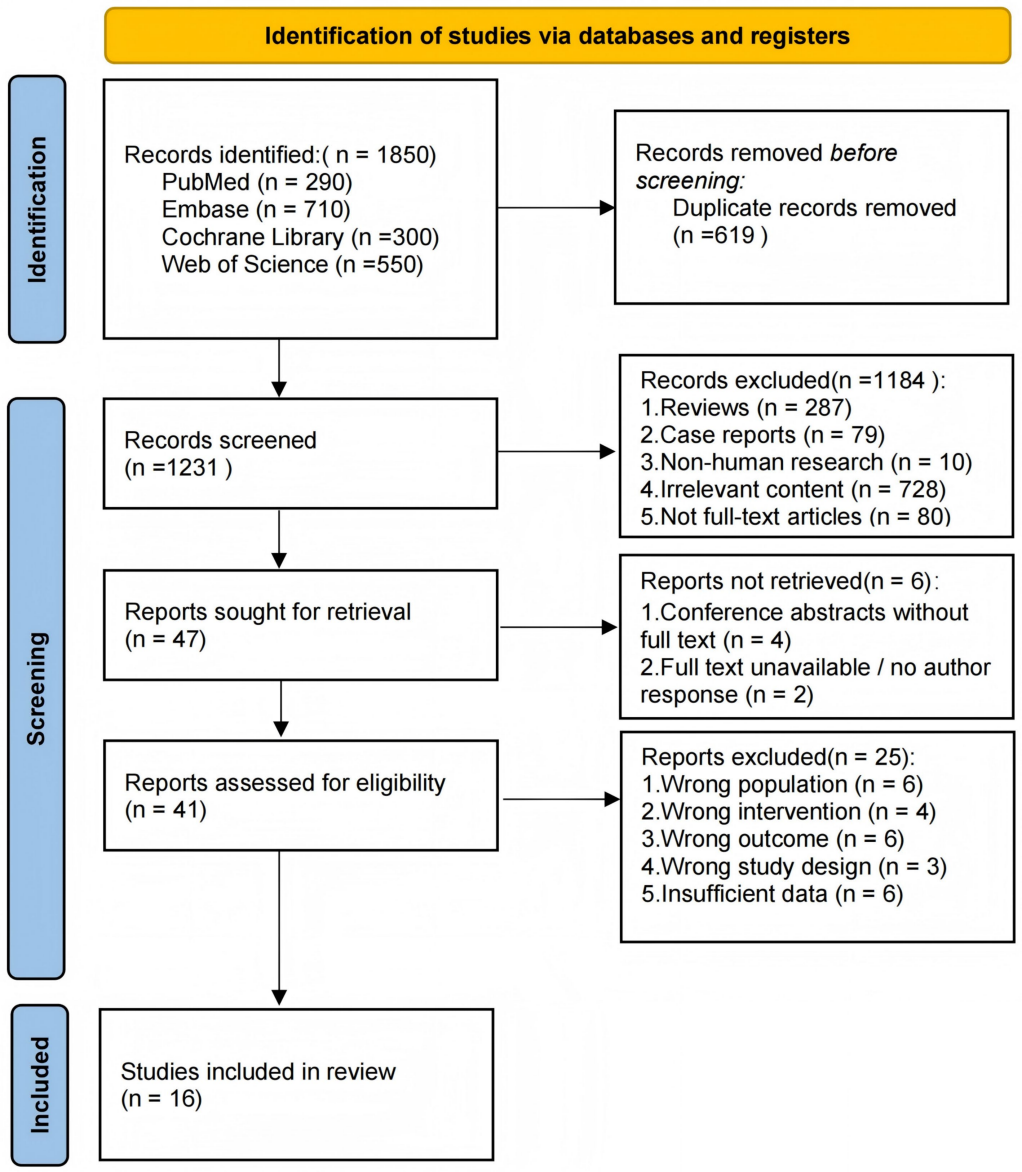
PRISMA flow diagram used in the study description.

A total of 1,850 records were initially identified through database searching. After removing 619 duplicates, 1,231 records remained. After screening titles and abstracts, 1,184 records were excluded, including 287 reviews, 79 case reports, 10 non-human studies, 728 articles with irrelevant content, and 80 non-full-text articles. The remaining 47 reports were sought for retrieval. Among these, six could not be obtained: four were conference abstracts without full text, and for two we attempted to contact the authors but could not obtain the data. The remaining 41 full-text articles were assessed for eligibility, and 25 were excluded for the following reasons: wrong population (*n* = 6), wrong intervention (*n* = 4), wrong outcome (*n* = 6), wrong study design (*n* = 3), or insufficient data (*n* = 6). Finally, 16 published randomized controlled trials met the inclusion criteria and were included in the systematic review and meta-analysis. These 16 studies comprised 414 participants. The study selection process is shown in the PRISMA flow diagram (Figure 1).

### 3.2 Characteristics of included studies

A total of 16 randomized controlled trials (RCTs), published in English between 2014 and 2024, enrolled 414 participants with PD. All active tDCS employed a current intensity of 2 mA with stimulation durations of 13, 15, 20, 25, and 30 min. Motor symptoms were assessed using the Unified Parkinson’s Disease Rating Scale Part III (UPDRS-III) in 12 RCTs (De Icco et al., 2022; Lee and Kim, 2021; Yotnuengnit et al., 2018; Pisano et al., 2024; Manor et al., 2021; Benninger et al., 2010; da Silva et al., 2018; Costa-Ribeiro et al., 2017; Manenti et al., 2016; Manenti et al., 2018; Na et al., 2022; Biundo et al., 2015). Functional mobility was measured using the Timed Up and Go (TUG) test in nine RCTs (Pisano et al., 2024; Manor et al., 2021; Costa-Ribeiro et al., 2016; Manenti et al., 2016; Na et al., 2022; Costa-Ribeiro et al., 2016; Wong et al., 2022; Wong et al., 2024; de Almeida et al., 2024). Detailed characteristics of all included studies are summarized in Table 1.

**Table 1:** Baseline characteristics of included studies.

Study	Country	Sample size (E/C)	Age (E/C, years)	Disease duration (E/C, years)	Stimulation site	Electrode size/current density	Stimulation Parameters	Stimulation duration	Concomitant therapy	Outcomes
De Icco et al. (2022)	Italy	13/11	71.90 ± 5.20; 73.70 ± 5.00	8.70 ± 5.80; 9.80 ± 8.80	Bi-hemispheric M1	9 cm^2^/0.22 mA/cm^2^	2 mA, 20 min	5 times/week for 1 week	Neurorehabilitation	UPDRS-III
Costa-Ribeiro et al. (2016)	Brazil	11/11	61.10 ± 9.10; 62.00 ± 16.70	–	SMA	35 cm^2^/0.06 mA/cm^2^	2 mA, 13 min	3 times/week for 4 weeks	Visually cued gait training	TUG
Lee and Kim (2021)	Korea	15/15	70.00 ± 3.76; 70.33 ± 3.27	6.27 ± 1.03; 6.67 ± 1.41	SMA	35 cm^2^/0.06 mA/cm^2^	2 mA, 20 min	5 times/week for 4 weeks	Visual cueing	UPDRS-III
Wong et al. (2022)	China	9/9	54.20 ± 4.10; 58.30 ± 8.00	7.80 ± 5.68; 8.35 ± 12.25	M1/DLPFC/Cerebellum	35 cm^2^/0.06 mA/cm^2^	2 mA, 20 min	1 session within 1 week	–	TUG
Yotnuengnit et al. (2018)	Thailand	17/18	68.20 ± 9.80; 62.70 ± 8.80	9.40 ± 5.30; 6.60 ± 3.60	Lower limb M1	35 cm^2^/0.06 mA/cm^2^	2 mA, 30 min	3 times/week for 2 weeks	Physical therapy	UPDRS-III
Pisano et al. (2024)	Italy	9/8	71.00 ± 8.60; 65.30 ± 8.50	–	Cerebellum	35 cm^2^/0.06 mA/cm^2^	2 mA, 20 min	Once daily for 10 days	C-Mill augmented reality treadmill	UPDRS-III /TUG
Manor et al. (2021)	USA	37/36	68.70 ± 8.20; 68.90 ± 7.40	10.40 ± 7.20; 9.20 ± 5.90	M1 + left DLPFC	–	2 mA, 20 min	5 times/week for 2 weeks	–	UPDRS-III/TUG
Benninger et al. (2010)	USA	13/12	63.60 ± 9.00; 64.20 ± 8.80	10.60 ± 7.10; 9.10 ± 3.30	M1 + prefrontal cortex	97.5 cm^2^/0.02 mA/cm^2^	2 mA, 20 min	3 times/week for 2.5 weeks	–	UPDRS-III
da Silva et al. (2018)	Brazil	8/9	66.00 ± 5.00; 66.00 ± 10.00	6.00 ± 6.00; 5.00 ± 1.00	SMA + medial M1	35 cm^2^/0.06 mA/cm^2^	2 mA, 15 min	Once daily for 10 days	Prior group-based exercise program	UPDRS-III
Costa-Ribeiro et al. (2017)	Brazil	11/11	61.10 ± 9.10; 62.00 ± 16.70	6.10 ± 3.80; 6.30 ± 3.70	SMA	35 cm^2^/0.06 mA/cm^2^	2 mA, 13 min	Once daily for 10 days	Cueing gait training with visual cues	UPDRS-III /TUG
Manenti et al. (2016)	Italy	10/10	69.00 ± 9.10; 69.10 ± 5.60	7.10 ± 3.60; 7.80 ± 4.20	DLPFC	35 cm^2^/0.06 mA/cm^2^	2 mA, 25 min	5 times a week for 2 weeks	Physical therapy	UPDRS-III/TUG
Manenti et al. (2018)	Italy	11/11	65.50 ± 6.40; 63.80 ± 7.10	7.20 ± 3.90; 7.60 ± 3.40	Left DLPFC	35 cm^2^/0.06 mA/cm^2^	2 mA, 25 min	5 times/week for 2 weeks	Treadmill training	UPDRS-III
Wong et al. (2024)	China	17/17	68.10 ± 5.80; 66.80 ± 6.90	5.50 ± 4.30; 7.90 ± 5.60	DLPFC	35 cm^2^/0.06 mA/cm^2^	2 mA, 20 min	2–3 times/week for 5 weeks	Treadmill training	TUG
Na et al. (2022)	Korea	11/12	63.73 ± 6.57; 65.08 ± 6.46	–	M1 leg	28.26 cm^2^/0.07 mA/cm^2^	2 mA, 20 min	10 sessions for 4 weeks	Treadmill gait training	UPDRS-III/TUG
Biundo et al. (2015)	Italy	7/9	69.10 ± 7.60; 72.30 ± 4.10	–	Left DLPFC	–	2 mA, 20 min	4 days/week for 4 weeks	Computerized cognitive training	UPDRS-III
de Almeida et al. (2024)	Brazil	8/8	69.62 ± 0.76; 64.00 ± 2.00	–	SMA	35 cm^2^/0.06 mA/cm^2^	2 mA, 20 min	5 times/week for 2 weeks	Exercise protocol	TUG

E/C, experimental/control; M1, primary motor cortex; DLPFC, dorsolateral prefrontal cortex; SMA, supplementary motor area; Bi-hemispheric M1, bilateral primary motor cortex stimulation; M1 leg, leg representation area of primary motor cortex; Lower limb M1, leg representation area of primary motor cortex; Left DLPFC, left dorsolateral prefrontal cortex; M1 + prefrontal cortex, primary motor cortex combined with prefrontal cortex; SMA + medial M1, supplementary motor area combined with medial primary motor cortex; UPDRS-III, Unified Parkinson’s Disease Rating Scale Part III; TUG, Timed Up and Go test; “–,” no relevant data obtained.

### 3.3 Risk of bias and GRADE evidence

**Figure 2:**
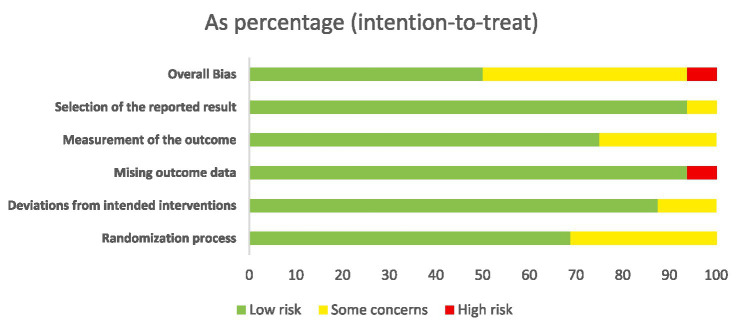
Risk of bias analysis of the included studies.

**Figure 3:**
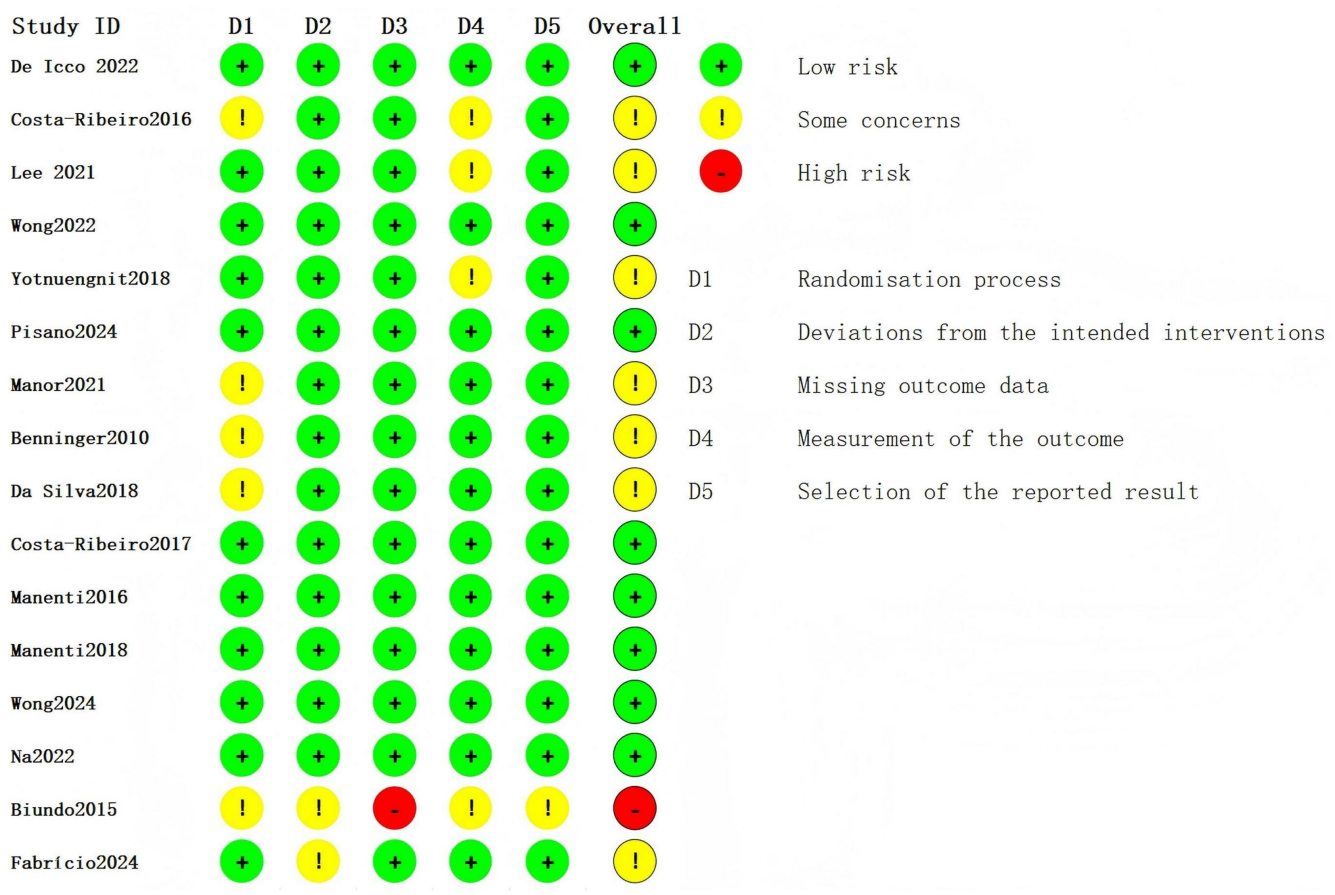
Risk of bias analysis of the included studies.

The risk of bias was evaluated for all 16 RCTs using ROB 2.0 tool, overall, 50% of the studies were rated as low risk, 43.8% had some concerns, and 6.3% were rated as high risk (Figure 2). Figure 3 presents the domain-level and overall risk-of-bias judgments for each study.

The 16 included studies showed a relatively low risk of methodological bias across four domains: (1) randomization process (11 studies with low risk, five with some concerns); (2) deviations from intended interventions (14 low risk, two some concerns); (3) measurement of the outcome (12 low risk, four some concerns); and (4) selection of the reported results (15 low risk, one some concerns). For the domain of missing outcome data, one study was judged to be at high risk of bias because it failed to address substantial missing outcome data appropriately.

The GRADE evidence grading showed that both UPDRS-III and TUG outcomes were rated as moderate quality. Downgrading was mainly due to risk of bias (see above) and imprecision (wide confidence intervals crossing the null, and insufficient sample size). Detailed domain-specific assessments are provided in Supplementary Table S2.

### 3.4 Pairwise meta-analysis

**Figure 4:**
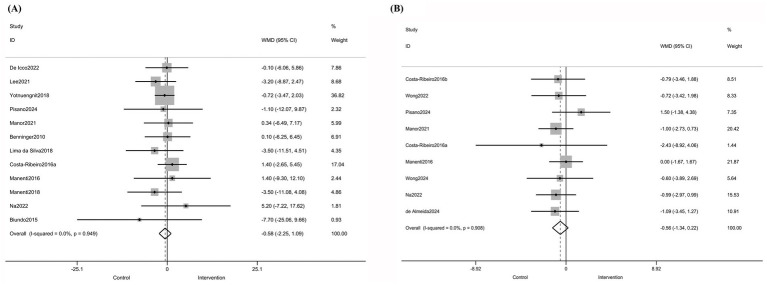
**(A)** Traditional pairwise meta-analysis of UPDRS-III score. **(B)** Traditional pairwise meta-analysis of TUG score.

The results of the traditional pairwise meta-analysis are presented in Figures 4A,B. A total of 16 studies were included in this systematic review, of which 12 assessed the effect of tDCS on UPDRS-III scores in patients with Parkinson’s disease [MD = −0.58, 95% CI (−2.25, 1.09), *I^2^* = 0.0%; *p* = 0.949]. Additionally, nine studies assessed the effect of tDCS on TUG scores [MD = −0.56, 95% CI (−1.34, 0.22), *I^2^* = 0.0%; *p* = 0.908]. Meta-analyses of the two primary outcome measures, UPDRS-III and TUG, showed no statistically significant heterogeneity across the included studies. Accordingly, data from all eligible participants were pooled in the final effect size calculations.

### 3.5 Network meta-analysis results

#### 3.5.1 Network plots

**Figure 5:**
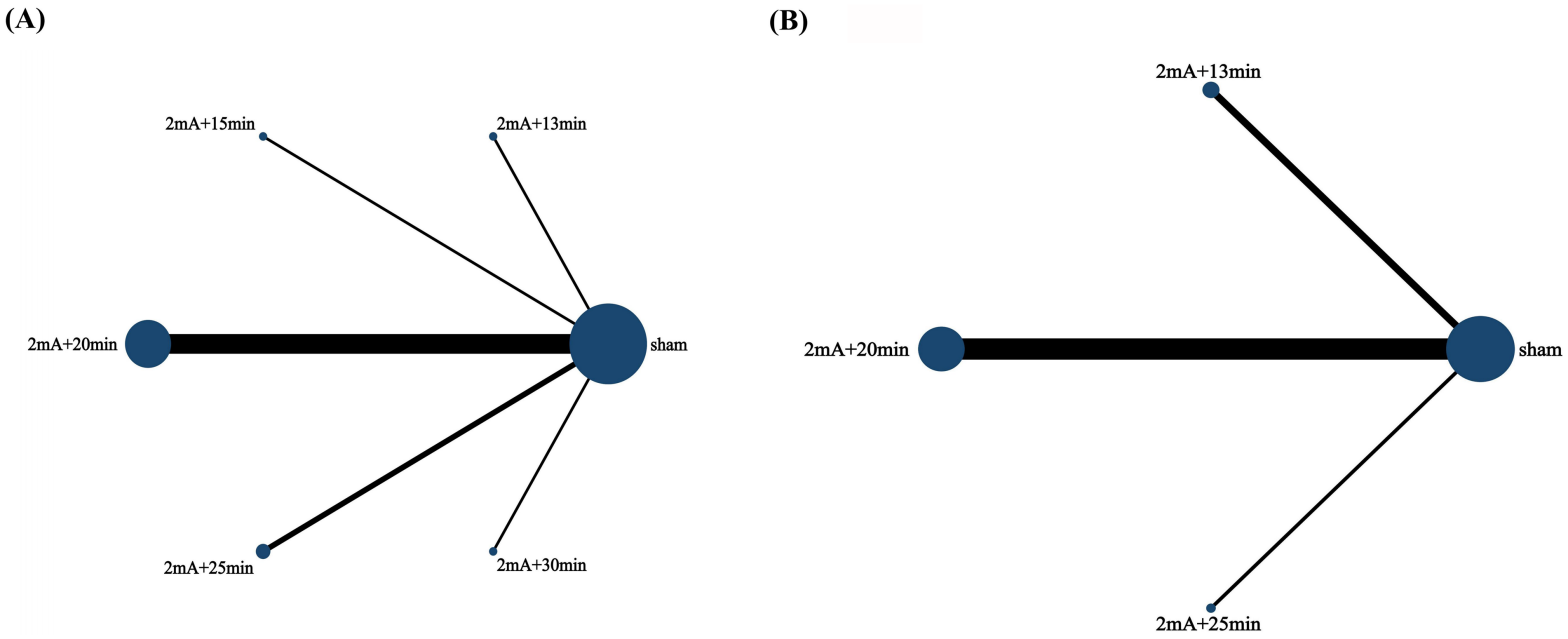
Network graph of direct comparisons. **(A)** UPDRS-III score; **(B)** TUG score. A line between two dots indicates a direct comparison between two interventions. Line thickness represents the number of studies directly comparing the two interventions, and dot size represents the sample size.

The UPDRS-III network (Figure 5A) contained six interventions and five direct comparisons. The TUG network (Figure 5B) evaluated four interventions and conducted three direct comparisons. All networks were open-loop (no closed loops), indicating that only direct comparisons between active tDCS and sham stimulation were available. The most frequently used tDCS dose was 2 mA + 20 min.

#### 3.5.2 Basic hypothesis testing

The tests for both models (with five and three dimensions) yielded non-significant results (*χ^2^* = 0.26, *p* = 0.608; *χ^2^* = 0.00, *p* = 1.000, respectively), confirming that the treatment effects across studies were consistent and that no substantial inconsistency was present in the direct evidence base.

#### 3.5.3 League table

Pairwise comparisons between active anodal tDCS of different durations (2 mA with stimulation durations of 13, 15, 20, 25, and 30 min) and sham stimulation are presented in Table 2A (UPDRS-III) and Table 2B (TUG). Results indicated that no active duration showed statistically significant superiority over sham for either motor (UPDRS-III) or mobility (TUG) outcomes, as all 95% confidence intervals included zero. Furthermore, direct comparisons between any two active tDCS durations revealed no statistically significant differences for either outcome measure. These results indicate that, within the tested duration range, no specific stimulation duration can be distinguished from sham or from any other active duration based on the current evidence.

**Table 2:** Network meta-analysis of various interventions: (A) UPDRS-III score and (B) TUG score.

(A)					
2 mA + 15 min	1.64 (−8.48,11.76)	2.74 (−5.76,11.24)	2.78 (−5.69,11.25)	3.50 (−4.51,11.51)	4.90 (−4.08,13.88)
−1.64 (−11.76, 8.48)	2 mA + 25 min	1.11 (−5.70,7.91)	1.14 (−5.62,7.91)	1.86 (−4.32,8.05)	3.26 (−4.13,10.65)
−2.74 (−11.24, 5.76)	−1.11 (−7.91, 5.70)	2 mA + 20 min	0.04 (−3.92,3.99)	0.76 (−2.09,3.60)	2.16 (−2.79,7.10)
−2.78 (−11.25, 5.69)	−1.14 (−7.91, 5.62)	−0.04 (−3.99, 3.92)	2 mA + 30 min	0.72 (−2.03,3.47)	2.12 (−2.77,7.01)
−3.50 (−11.51, 4.51)	−1.86 (−8.05, 4.32)	−0.76 (−3.60, 2.09)	−0.72 (−3.47, 2.03)	sham	1.40 (−2.65,5.45)
−4.90 (−13.88, 4.08)	−3.26 (−10.65, 4.13)	−2.16 (−7.10, 2.79)	−2.12 (−7.01, 2.77)	−1.40 (−5.45, 2.65)	2 mA + 13 min

(B)			
2 mA + 13 min	0.35 (−2.29,3.00)	1.03 (−1.95,4.01)	1.03 (−1.44,3.50)
−0.35 (−3.00, 2.29)	2 mA + 20 min	0.68 (−1.24,2.59)	0.68 (−0.27,1.62)
−1.03 (−4.01, 1.95)	−0.68 (−2.59, 1.24)	2 mA + 25 min	−0.00 (−1.67,1.67)
−1.03 (−3.50, 1.44)	−0.68 (−1.62, 0.27)	0.00 (−1.67, 1.67)	sham

#### 3.5.4 Ranking of intervention efficacy

**Figure 6:**
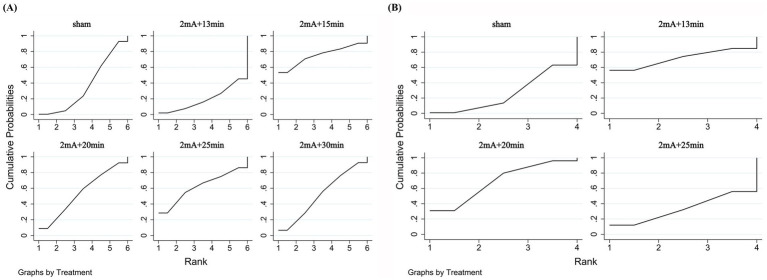
Cumulative probability of each intervention. A higher SUCRA value indicates a better rank **(A)** UPDRS-III score; **(B)** TUG score.

The 2 mA + 15 min tDCS had the highest probability of being optimal for improving UPDRS-III (Figure 6A), and the 2 mA + 13 min tDCS was likely to be optimal for reducing TUG time (Figure 6B) relative to other interventions. However, these patterns lack statistical support and may simply reflect sampling variability. Because no pairwise comparison between active durations reached statistical significance and the network was sparse, the SUCRA rankings are presented as exploratory only and should not be interpreted as evidence of a true efficacy hierarchy.

### 3.6 Publication bias analysis

**Figure 7:**
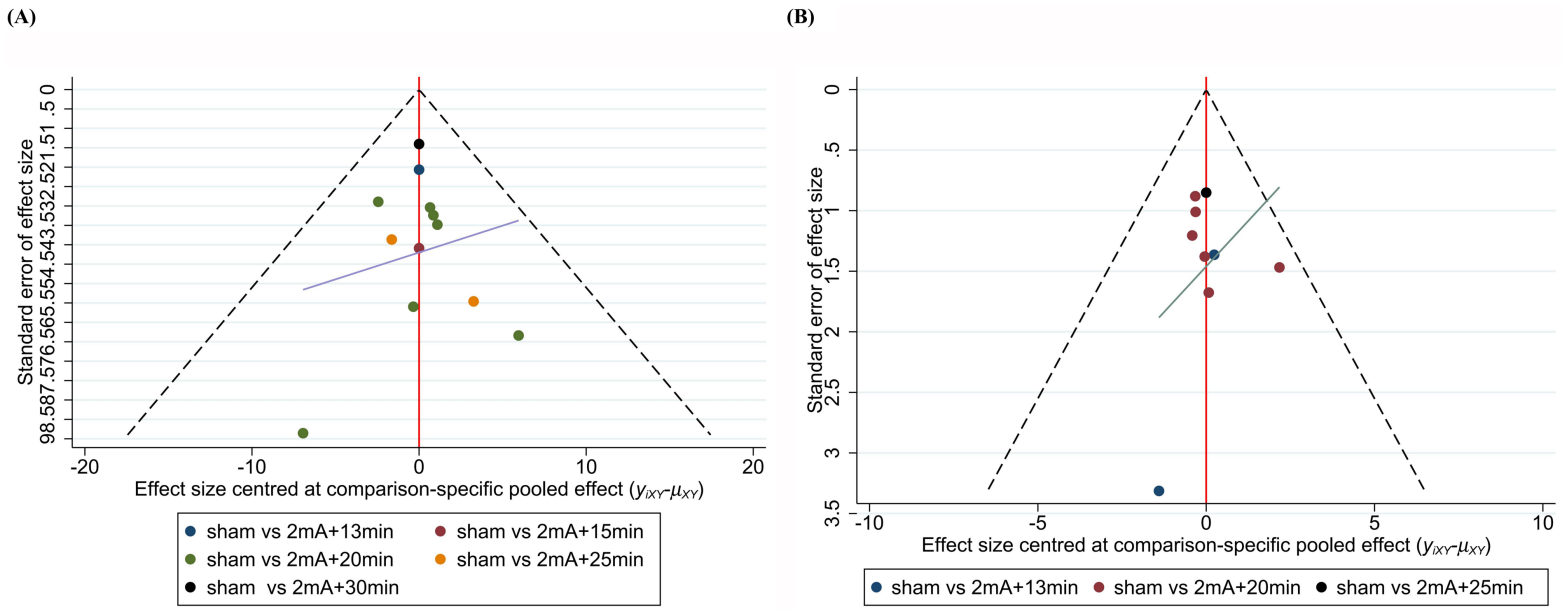
Funnel plots for **(A)** UPDRS-III score and **(B)** TUG score.

Funnel plots for UPDRS-III (Figure 7A) and TUG (Figure 7B) were roughly symmetrical. Egger’s tests yielded *p* = 0.631 (>0.05) for UPDRS-III and *p* = 0.437 (>0.05) for TUG, indicating no clear publication bias.

## 4 Discussion

### 4.1 Summary of findings

The present network meta-analysis demonstrated no significant differences in UPDRS-III or TUG outcomes among all active tDCS durations (2 mA with stimulation durations of 13, 15, 20, 25, and 30 min) compared to sham, nor between any two active durations. However, according to the SUCRA ranking, 2 mA + 15 min tDCS may be optimal for improving UPDRS-III, and 2 mA + 13 min tDCS may be optimal for reducing TUG time. Nevertheless, these patterns lack statistical support and may simply reflect sampling variability. Because no pairwise comparison between active durations reached statistical significance and the network was sparse, the SUCRA rankings are presented as exploratory only and should not be interpreted as evidence of a true efficacy hierarchy. This study is the first network meta-analysis to simultaneously compare multiple single-session tDCS durations for motor symptoms in PD. Thus, the null findings should not be interpreted as definitive proof that stimulation duration is irrelevant; instead, they highlight substantial evidence gaps, and the observed lack of statistical superiority likely reflects these limitations rather than a true absence of differential effects.

### 4.2 Potential mechanistic interpretation

SUCRA rankings showed that 2 mA + 15 min tDCS may be optimal for improving UPDRS-III, whereas 2 mA + 13 min tDCS may be optimal for shortening TUG time. One might speculate that longer durations preferentially modulate motor circuits, while shorter durations affect prefrontal-striatal circuits (Bolognini et al., 2009; Borda et al., 2022; Yogev-Seligmann et al., 2008). However, such mechanistic interpretation is highly speculative and not supported by the current data. The sparse network, lack of significant direct comparisons, and wide confidence intervals preclude any firm conclusion about circuit-specific effects.

Previous studies have suggested potential mechanisms such as LTP-like plasticity in motor cortex (Bolognini et al., 2009), transient desynchronization of prefrontal-striatal circuits (Polanía et al., 2011; Monte-Silva et al., 2010; Hong et al., 2022), and neural adaptation or synaptic fatigue with prolonged stimulation (Jamil et al., 2017). Interindividual variability in neuroanatomical and neurochemical factors may further modulate responses (Gonzalez-Escamilla et al., 2022; Dayan et al., 2013). Nevertheless, these remain speculative hypotheses that require direct validation.

Therefore, the present rankings should be viewed only as hypothesis-generating observations. Future studies integrating neuroimaging or electrophysiology are needed to directly test whether duration-dependent modulation of distinct neural networks exists. Until then, no mechanistic claims can be made.

### 4.3 Comparison with existing literature

The optimal stimulation parameters of tDCS for PD remain clinically controversial. Previous studies confirmed that tDCS improves gait and balance in PD (Lee et al., 2025; de Oliveira et al., 2021; Elsner et al., 2016; Goodwill et al., 2017; Nascimento et al., 2021; Lefaucheur et al., 2017). However, no consensus exists on key variables such as stimulation duration. Prior systematic reviews noted that substantial variations in parameters (e.g., current intensity, target site, number of sessions) lead to considerable heterogeneity, making it difficult to derive a universal optimal set (Liu et al., 2021; Pol et al., 2021; de Oliveira et al., 2021; Domínguez-Pera et al., 2025; Fregni et al., 2005).

The present network meta-analysis specifically examined differential effects of stimulation duration on functional outcomes. Although early reviews [e.g., Beretta et al. (2022)] indicated that tDCS improves balance, their conclusions were based on highly heterogeneous protocols. Our analysis reveals that this apparent “protocol insensitivity” may mask parameter requirements that depend on distinct functional networks. We identified divergent trends: enhancing core motor function (UPDRS-III) favored 2 mA + 15 min tDCS, whereas improving comprehensive mobility (TUG) favored 2 mA + 13 min tDCS. This aligns with the “function-specific optimization” principle, suggesting that different tasks rely on different circuits and may require differentiated plasticity windows (Polanía et al., 2018; Stagg and Nitsche, 2011). Therefore, rather than advocating a single “one-size-fits-all” optimal duration, our results support a stratified benefit model: different functional domains may benefit from different stimulation windows.

Although these duration differences did not reach statistical significance (Li et al., 2015; Wiethoff et al., 2014). The trend pattern addresses the call for systematic investigation of nonlinear dose–response relationships (Lewis et al., 2025; Woods et al., 2016). Accordingly, the primary contribution of this study is challenging the conventional search for universal “optimal parameters” (Bikson et al., 2018). We propose that parameter optimization is goal-directed and function-specific, grounded in neural circuits underlying rehabilitation objectives (Polanía et al., 2018). This work shifts from seeking a “single optimal solution” to establishing an “outcome-based hierarchy of parameter effects,” offering a new framework to interpret prior heterogeneity and design future precision trials (Broeder et al., 2015). Future research should adopt multi-domain strategies and integrate predictive biomarkers (e.g., neuroimaging features, genetic background) (Li et al., 2015; Filmer et al., 2014) to validate this concept in larger studies.

### 4.4 Clinical significance

We further evaluated the clinical relevance of the observed effect sizes. The pooled mean difference for UPDRS-III was −0.58, substantially smaller than the minimal clinically important difference (MCID) of −3.25 points (Horváth et al., 2015). For TUG, the pooled effect was −0.56 s, but no validated PD-specific MCID for the single-task TUG is available. Thus, even if these effects were statistically significant, they would lack clinical relevance.

Clinicians should interpret the null findings with caution: absence of statistical significance does not prove equivalence, and the observed numerical trends should not guide clinical decision-making without further validation. Previous research suggests that tDCS combined with motor-cognitive training may yield larger clinical benefits. For example, cerebellar tDCS with augmented reality treadmill training improved freezing of gait (Pisano et al., 2024), and tDCS prolonged the benefits of cueing gait training on functional mobility (Costa-Ribeiro et al., 2017). Future trials should directly compare standalone tDCS with such combination protocols to identify strategies that produce clinically meaningful improvements.

### 4.5 Limitations and future directions

This study has several limitations. First, the network meta-analysis is underpowered. Most included studies had small sample sizes, and most comparisons relied on single studies with wide confidence intervals, implying that only large effect sizes were detectable; smaller, yet potentially clinically meaningful differences could not be ruled out. Therefore, the null findings may reflect insufficient power rather than a true absence of duration effects. Current evidence is insufficient to draw definitive conclusions. Second, the analysis mixed single- and multi-session studies, using per-session duration as the sole treatment node, which may violate transitivity. The total number of tDCS sessions across included studies ranged from 1 to 20 sessions. Given the wide variation in total session counts and the generally small sample size of individual trials, the comparability between different treatment nodes is limited, which further compromises the robustness of our findings. Hence, the findings on per-session duration are exploratory, and head-to-head trials are needed to separate single- from multi-session effects. Third, inconsistent reporting of medication status may introduce unmeasured heterogeneity, and stratification was not feasible. The observed *I^2^* = 0% may reflect low power rather than true homogeneity. Thus, pooling on- and off-medication scores remains a limitation, and null findings should be interpreted with caution. Fourth, grey literature was not searched, which may have introduced selection bias. Fifth, the analysis is based on a limited number of small-sample studies, and the probability rankings are likely influenced by sampling variability. Future multicenter, large-sample RCTs with standardized tDCS protocols are needed to determine whether specific durations (e.g., 2 mA + 13 min tDCS, 2 mA + 15 min tDCS) confer clinically meaningful benefits. Finally, based on the core finding that different functional domains require different optimal durations, future research should adopt more targeted approaches. A priority is a multi-group RCT directly comparing 2 mA + 13 min tDCS, 2 mA + 15 min tDCS, and sham stimulation, using a common set of primary outcomes to evaluate pure motor and integrated cognitive-motor function. This design will confirm the observed dissociation. Research should also extend to other current intensities (e.g., 1 mA, 1.5 mA) to test whether optimal durations remain stable. Lastly, prospective collection of individual factors (e.g., cortical anatomy, baseline dopamine levels) is essential for developing predictive models and personalized tDCS for Parkinson’s disease.

## 5 Conclusion

This network meta-analysis shows that no single-session tDCS duration (2 mA with stimulation durations of 13, 15, 20, 25, and 30 min) is statistically superior to sham for improving motor function in PD. Although probability rankings suggest that 2 mA + 15 min tDCS may be optimal for UPDRS-III and 2 mA + 13 min tDCS for TUG, these rankings are descriptive only owing to sparse indirect evidence and should not be interpreted as a definitive efficacy hierarchy. The observed numerical improvements (0.35–1.03 s) did not reach statistical significance, and the effect sizes are below clinically meaningful thresholds. Therefore, based on current limited evidence, no specific stimulation duration can be recommended as superior. Varying stimulation time alone within this range does not decisively impact outcomes. Our findings highlight the need for multidimensional optimization of tDCS and further high-quality, large-sample RCTs to determine whether any duration confers clinically meaningful benefits.

## Data Availability

The original contributions presented in the study are included in the article/Supplementary material, further inquiries can be directed to the corresponding author.
