# Supplementary material for: The different stimulation durations of transcranial direct current stimulation for Parkinson’s disease: a systematic review and network meta-analysis
Source: Front Aging Neurosci. 2026 Jun 10;18:1849992. doi: 10.3389/fnagi.2026.1849992 (PMC13290960; doi:10.3389/fnagi.2026.1849992)
Supplement: Supplementary file 2 [file Table_1.docx]

Supplementary Material

# Supplementary Data

1. **Supplementary Tables**

**Supplementary Table 1**Search strategy in PubMed

| Search number | Query | Search Details | Results |
| --- | --- | --- | --- |
| 1 | Parkinson Disease | "Parkinson Disease"[Mesh] | 87,880 |
| 2 | "idiopathic parkinsonism" OR "Idiopathic Parkinson's Disease" OR "Lewy bodies of Parkinson disease" OR "Lewy bodies of Parkinsons disease" OR "Lewy body Parkinson disease" OR "Lewy body Parkinsons disease" OR "Lewy Body Parkinson's Disease" OR "paralysis agitans" OR "Parkinson dementia complex" OR "Parkinsonism" OR "Parkinson's Disease" OR "primary parkinsonism" | "idiopathic parkinsonism"[Title/Abstract] OR "Idiopathic Parkinson's Disease"[Title/Abstract] OR "Lewy bodies of Parkinson disease"[Title/Abstract] OR "Lewy bodies of Parkinsons disease"[Title/Abstract] OR "Lewy body Parkinson disease"[Title/Abstract] OR "Lewy body Parkinsons disease"[Title/Abstract] OR "Lewy Body Parkinson's Disease"[Title/Abstract] OR "paralysis agitans"[Title/Abstract] OR "Parkinson dementia complex"[Title/Abstract] OR "Parkinsonism"[Title/Abstract] OR "Parkinson's Disease"[Title/Abstract] OR "primary parkinsonism"[Title/Abstract] | 131,515 |
| 3 | Transcranial Direct Current Stimulation | "Transcranial Direct Current Stimulation"[Mesh] | 5,726 |
| 4 | "anodal stimulation tdcs" OR "anodal stimulation tdcs" OR "anodal stimulation transcranial direct current stimulatio" OR "cathodal stimulation tdcs" OR "cathodal stimulation tdcs" OR "cathodal stimulation transcranial direct current stimulation" OR "Repetitive Transcranial Electrical Stimulation" OR "tdcs" OR "transcranial alternating current stimulation" OR "transcranial electrical stimulation" OR "transcranial electrical stimulations" OR "transcranial random noise stimulation" | "anodal stimulation tdcs"[Title/Abstract] OR "anodal stimulation tdcs"[Title/Abstract] OR "anodal stimulation transcranial direct current stimulatio"[Title/Abstract] OR "cathodal stimulation tdcs"[Title/Abstract] OR "cathodal stimulation tdcs"[Title/Abstract] OR "cathodal stimulation transcranial direct current stimulation"[Title/Abstract] OR "Repetitive Transcranial Electrical Stimulation"[Title/Abstract] OR "tdcs"[Title/Abstract] OR "transcranial alternating current stimulation"[Title/Abstract] OR "transcranial electrical stimulation"[Title/Abstract] OR "transcranial electrical stimulations"[Title/Abstract] OR "transcranial random noise stimulation"[Title/Abstract] | 9,120 |
| 5 | (#1 OR #2) AND (#3 OR #4) | (("Parkinson Disease"[Mesh]) OR ("idiopathic parkinsonism"[Title/Abstract] OR "Idiopathic Parkinson's Disease"[Title/Abstract] OR "Lewy bodies of Parkinson disease"[Title/Abstract] OR "Lewy bodies of Parkinsons disease"[Title/Abstract] OR "Lewy body Parkinson disease"[Title/Abstract] OR "Lewy body Parkinsons disease"[Title/Abstract] OR "Lewy Body Parkinson's Disease"[Title/Abstract] OR "paralysis agitans"[Title/Abstract] OR "Parkinson dementia complex"[Title/Abstract] OR "Parkinsonism"[Title/Abstract] OR "Parkinson's Disease"[Title/Abstract] OR "primary parkinsonism"[Title/Abstract])) AND (("Transcranial Direct Current Stimulation"[Mesh]) OR ("anodal stimulation tdcs"[Title/Abstract] OR "anodal stimulation tdcs"[Title/Abstract] OR "anodal stimulation transcranial direct current stimulatio"[Title/Abstract] OR "cathodal stimulation tdcs"[Title/Abstract] OR "cathodal stimulation tdcs"[Title/Abstract] OR "cathodal stimulation transcranial direct current stimulation"[Title/Abstract] OR "Repetitive Transcranial Electrical Stimulation"[Title/Abstract] OR "tdcs"[Title/Abstract] OR "transcranial alternating current stimulation"[Title/Abstract] OR "transcranial electrical stimulation"[Title/Abstract] OR "transcranial electrical stimulations"[Title/Abstract] OR "transcranial random noise stimulation"[Title/Abstract])) | 290 |

**Search strategy in Embase**

| Search number | Query | Results |
| --- | --- | --- |
| 1 | parkinson disease'/exp | 210,761 |
| 2 | idiopathic parkinson disease':ti,ab,kw OR 'idiopathic parkinsonism':ti,ab,kw OR 'idiopathic parkinsons disease':ti,ab,kw OR 'lewy bodies of parkinson disease':ti,ab,kw OR 'lewy bodies of parkinsons disease':ab,ti OR 'lewy body parkinson disease':ti,ab,kw OR 'lewy body parkinsons disease':ti,ab,kw OR 'paralysis agitans':ti,ab,kw OR 'parkinson dementia complex':ti,ab,kw OR parkinsonism:ti,ab,kw OR 'parkinsons disease':ti,ab,kw OR 'primary parkinsonism':ti,ab,kw | 33,634 |
| 3 | trancranial direct current stimulation'/exp | 12,665 |
| 4 | anodal stimulation tdcs':ti,ab,kw OR 'anodal stimulation tdcss':ti,ab,kw OR 'anodal stimulation transcranial direct current stimulation':ti,ab,kw OR 'cathodal stimulation tdcs':ti,ab,kw OR 'cathodal stimulation tdcss':ti,ab,kw OR 'cathodal stimulation transcranial direct current stimulation':ti,ab,kw OR 'repetitive transcranial electrical stimulation':ti,ab,kw OR 'tdcs':ti,ab,kw OR 'transcranial alternating current stimulation':ti,ab,kw OR 'transcranial electrical stimulation':ti,ab,kw OR 'transcranial electrical stimulations':ti,ab,kw OR 'transcranial random noise stimulation':ti,ab,kw | 13,103 |
| 5 | (#1 OR #2) AND (#3 OR #4) | 710 |

**Search strategy in The Cochrane Library**

| Search number | Query | Search Details | Results |
| --- | --- | --- | --- |
| 1 | Parkinson Disease | [Parkinson Disease] explode all trees | 6,256 |
| 2 | "idiopathic parkinsonism" OR "Idiopathic Parkinson's Disease" OR "Lewy bodies of Parkinson disease" OR "Lewy bodies of Parkinsons disease" OR "Lewy body Parkinson disease" OR "Lewy body Parkinsons disease" OR "Lewy Body Parkinson's Disease" OR "paralysis agitans" OR "Parkinson dementia complex" OR "Parkinsonism" OR "Parkinson's Disease" OR "primary parkinsonism" | ("idiopathic parkinsonism" OR "Idiopathic Parkinson's Disease" OR "Lewy bodies of Parkinson disease" OR "Lewy bodies of Parkinsons disease" OR "Lewy body Parkinson disease" OR "Lewy body Parkinsons disease" OR "Lewy Body Parkinson's Disease" OR "paralysis agitans" OR "Parkinson dementia complex" OR "Parkinsonism" OR "Parkinson's Disease" OR "primary parkinsonism"):ti,ab,kw | 14410 |
| 3 | Transcranial Direct Current Stimulation | [Transcranial Direct Current Stimulation] explode all trees | 2064 |
| 4 | "anodal stimulation tdcs" OR "anodal stimulation tdcs" OR "anodal stimulation transcranial direct current stimulatio" OR "cathodal stimulation tdcs" OR "cathodal stimulation tdcs" OR "cathodal stimulation transcranial direct current stimulation" OR "Repetitive Transcranial Electrical Stimulation" OR "tdcs" OR "transcranial alternating current stimulation" OR "transcranial electrical stimulation" OR "transcranial electrical stimulations" OR "transcranial random noise stimulation" | ("anodal stimulation tdcs" OR "anodal stimulation tdcs" OR "anodal stimulation transcranial direct current stimulatio" OR "cathodal stimulation tdcs" OR "cathodal stimulation tdcs" OR "cathodal stimulation transcranial direct current stimulation" OR "Repetitive Transcranial Electrical Stimulation" OR "tdcs" OR "transcranial alternating current stimulation" OR "transcranial electrical stimulation" OR "transcranial electrical stimulations" OR "transcranial random noise stimulation" ):ti,ab,kw | 6726 |
| 5 | (#1 OR #2) AND (#3 OR #4) |  | 300 |

**Search strategy in Web of Science**

| Search number | Query | Results |
| --- | --- | --- |
| 1 | "parkinson disease" OR "idiopathic parkinsonism" OR "Idiopathic Parkinson's Disease" OR "Lewy bodies of Parkinson disease" OR "Lewy bodies of Parkinsons disease" OR "Lewy body Parkinson disease" OR "Lewy body Parkinsons disease" OR "Lewy Body Parkinson's Disease" OR "paralysis agitans" OR "Parkinson dementia complex" OR "Parkinsonism" OR "Parkinson's Disease" OR "primary parkinsonism" | 344,573 |
| 2 | "transcranial direct current stimulation" OR "anodal stimulation tdcs" OR "anodal stimulation tdcs" OR "anodal stimulation transcranial direct current stimulatio" OR "cathodal stimulation tdcs" OR "cathodal stimulation tdcs" OR "cathodal stimulation transcranial direct current stimulation" OR "Repetitive Transcranial Electrical Stimulation" OR "tdcs" OR "transcranial alternating current stimulation" OR "transcranial electrical stimulation" OR "transcranial electrical stimulations" OR "transcranial random noise stimulation" | 18,545 |
| 3 | #1 AND #2 | 550 |
