# Supplementary material for: The different stimulation durations of transcranial direct current stimulation for Parkinson’s disease: a systematic review and network meta-analysis
Source: Front Aging Neurosci. 2026 Jun 10;18:1849992. doi: 10.3389/fnagi.2026.1849992 (PMC13290960; doi:10.3389/fnagi.2026.1849992)
Supplement: Supplementary file 3 [file Table_2.pdf]

Table S2. GRADE certainty of evidence for network comparisons

Outcome	Participants (studies)	Effect Estimate (MD, 95% CI)	Risk of bias	Inconsistency	Indirectness	Imprecision	Publication bias	Overall Certainty Of evidence
UPDRS-III	324(12 RCTs)	-0.58 (-2.25,1.09)	Serious [a]	Not serious	Not serious	Serious [b]	Not serious	moderate
TUG	245(9 RCTs)	-0.56 (-1.34,0.22)	serious [a]	Not serious	Not serious	Serious [b]	Not serious	moderate

Notes:
RCT = randomized controlled trials.
a:Allocation concealment and blinding were subject to substantial bias
b:The sample size was small, leading to wide confidence intervals.
